# AI-Enhanced Virtual Reality Self-Talk for Psychological Counseling: Formative Qualitative Study

**DOI:** 10.2196/67782

**Published:** 2025-04-02

**Authors:** ‪Moreah Zisquit, Alon Shoa, Ramon Oliva, Stav Perry, Bernhard Spanlang, Anat Brunstein Klomek, Mel Slater, Doron Friedman

**Affiliations:** 1 Baruch Ivcher School of Psychology Reichman University Herzliya Israel; 2 Sammy Ofer School of Communications Reichman University Herzliya Israel; 3 Event Lab University of Barcelona Barcelona Spain; 4 Kiin S.L. Barcelona Spain

**Keywords:** virtual human, large language model, virtual reality, self-talk, psychotherapy, artificial intelligence, AI

## Abstract

**Background:**

Access to mental health services continues to pose a global challenge, with current services often unable to meet the growing demand. This has sparked interest in conversational artificial intelligence (AI) agents as potential solutions. Despite this, the development of a reliable virtual therapist remains challenging, and the feasibility of AI fulfilling this sensitive role is still uncertain. One promising approach involves using AI agents for psychological self-talk, particularly within virtual reality (VR) environments. Self-talk in VR allows externalizing self-conversation by enabling individuals to embody avatars representing themselves as both patient and counselor, thus enhancing cognitive flexibility and problem-solving abilities. However, participants sometimes experience difficulties progressing in sessions, which is where AI could offer guidance and support.

**Objective:**

This formative study aims to assess the challenges and advantages of integrating an AI agent into self-talk in VR for psychological counseling, focusing on user experience and the potential role of AI in supporting self-reflection, problem-solving, and positive behavioral change.

**Methods:**

We carried out an iterative design and development of a system and protocol integrating large language models (LLMs) within VR self-talk during the first two and a half years. The design process addressed user interface, speech-to-text functionalities, fine-tuning the LLMs, and prompt engineering. Upon completion of the design process, we conducted a 3-month long exploratory qualitative study in which 11 healthy participants completed a session that included identifying a problem they wanted to address, attempting to address this problem using self-talk in VR, and then continuing self-talk in VR but this time with the assistance of an LLM-based virtual human. The sessions were carried out with a trained clinical psychologist and followed by semistructured interviews. We used applied thematic analysis after the interviews to code and develop key themes for the participants that addressed our research objective.

**Results:**

In total, 4 themes were identified regarding the quality of advice, the potential advantages of human-AI collaboration in self-help, the believability of the virtual human, and user preferences for avatars in the scenario. The participants rated their desire to engage in additional such sessions at 8.3 out of 10, and more than half of the respondents indicated that they preferred using VR self-talk with AI rather than without it. On average, the usefulness of the session was rated 6.9 (SD 0.54), and the degree to which it helped solve their problem was rated 6.1 (SD 1.58). Participants specifically noted that human-AI collaboration led to improved outcomes and facilitated more positive thought processes, thereby enhancing self-reflection and problem-solving abilities.

**Conclusions:**

This exploratory study suggests that the VR self-talk paradigm can be enhanced by LLM-based agents and presents the ways to achieve this, potential pitfalls, and additional insights.

## Introduction

### Background

#### Overview

Access to mental health services is a critical issue on a global scale. Major depression is the leading cause of living with disability for many years and the fourth leading cause of disability-adjusted life years worldwide [1]. Furthermore, research indicates that >20% of people will experience mental illness in their lifetime [2]. Unfortunately, the current clinical workforce is insufficient, with approximately 9 psychiatrists per 100,000 people in high-income countries [3]. This shortfall in care has prompted interest in technology as a potential solution to bridge the gap between the need for treatment and the capacity to deliver it, particularly through the development of conversational agents or multipurpose virtual assistants [4]. These artificial intelligence (AI) agents can be integrated into virtual reality (VR) to simulate a personal, realistic therapeutic environment to reduce feelings of unnaturalness for the patient.

However, creating a virtual therapist presents significant challenges. Despite advancements in generative AI, the reliability of AI in sensitive therapeutic roles remains uncertain [4]. Some researchers argue that AI-based therapists should be used as tools to support both patients and therapists rather than serving as direct replacements for human therapists [5]. Building on this perspective, we propose the integration of an AI agent into the VR counseling experience as explored by Osimo et al [6] and Slater et al [7]. In this framework, participants embody 2 avatars: one representing themselves as a patient and the other as a counselor, allowing an “outside-looking-in” perspective that facilitates self-directed advice and reflection. However, sometimes self-conversation can become stuck, and the patient needs outside help. We contend that large language models (LLMs), while not yet fully capable of replacing human therapists, can function in this more limited role as an effective aid to help the conversation along, thereby enriching the self-talk process. This paper details the integration of an LLM-based agent into the VR self-talk paradigm. We outline the iterative design and development process, share the lessons learned, and present the first successful version, culminating in a small qualitative study in the context of motivational interviewing [8].

#### Self-Talk in VR

In times of crisis, individuals may find that their capacity to analyze issues and help themselves is limited. Their ability to gain insight is constrained, and it often feels like there is only one solution or, in more dire circumstances, “no way out.” Interestingly, contemplating the problem as an onlooker or from a friend’s perspective can improve problem-solving capabilities, a phenomenon known as Solomon’s paradox [9]. The paradox states that people reason more wisely regarding other people’s problems than they do about their own. Furthermore, a straightforward linguistic change from using “me or I” to “you” has also been shown to increase the psychological distance from personal problems, consequently alleviating the distress they cause and fostering cognitive flexibility [10]. Hence, a relatively straightforward shift in language and perception can enhance problem-solving capabilities.

**Figure 1:**
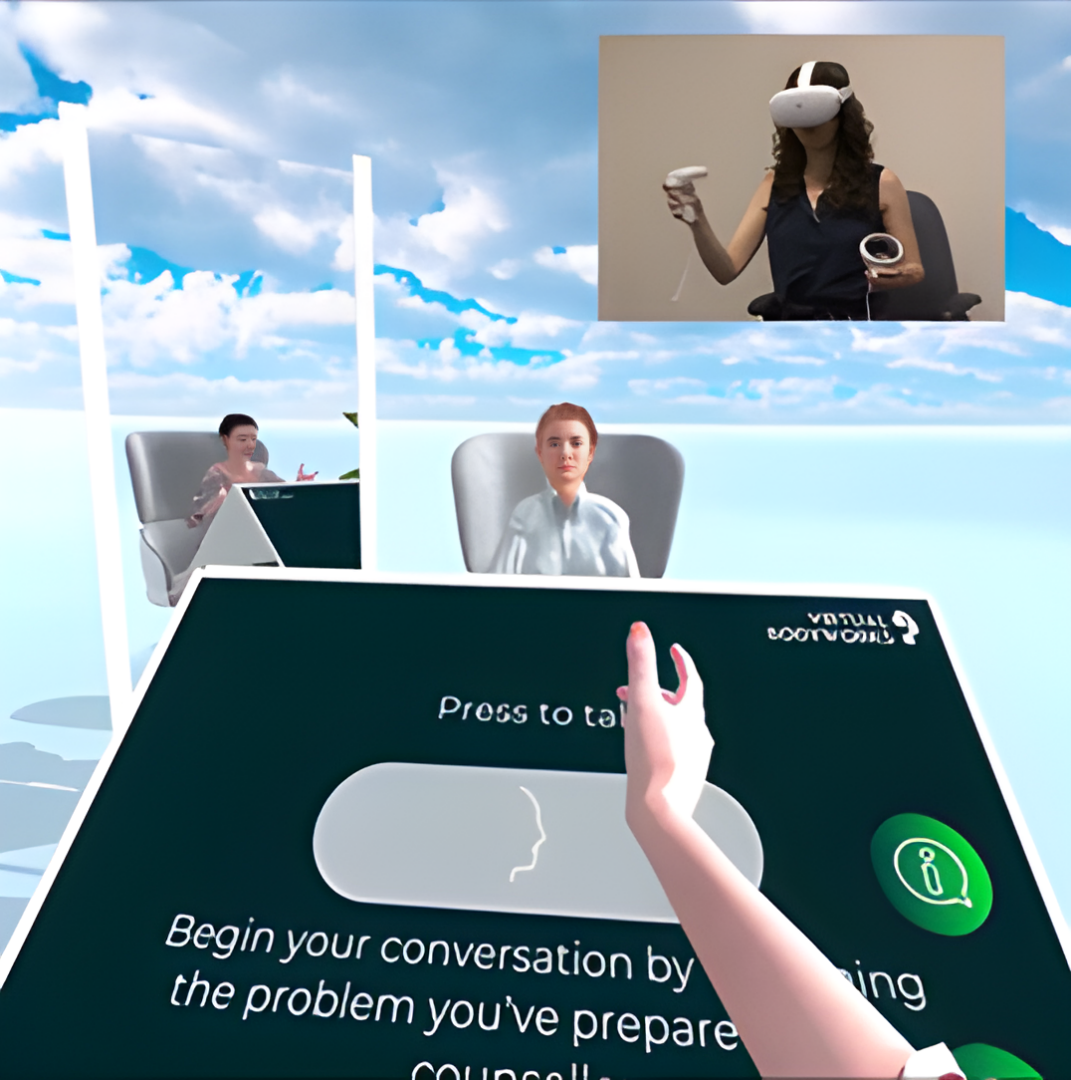
Screenshot of the counseling scenario interface. Top right inset shows a woman wearing a virtual reality headset (not a study participant). She is embodied as a female avatar reflected in a virtual mirror, with a same-sex counselor avatar seated across from her. A virtual control panel in front of her gives her instructions regarding how to proceed with the self-counseling experience and features an artificial intelligence help button.

**Figure 2:**
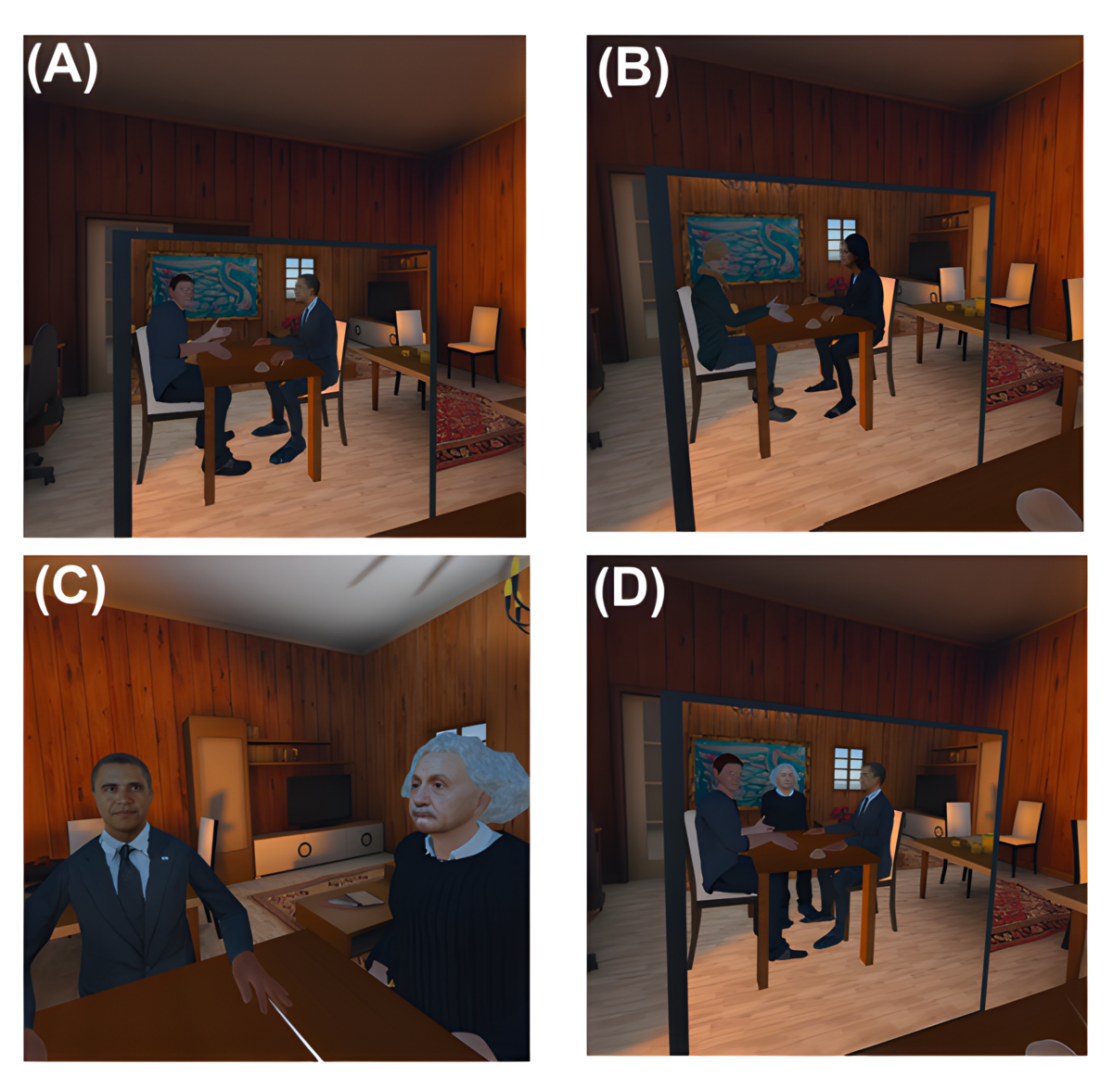
Screenshots from the counseling scenario. (A and B) Self-talk virtual reality (VR) as seen through the virtual mirror, featuring male and female avatars seated across from Barack Obama and Michelle Obama avatars, accordingly (both depicting the first VR experience without artificial intelligence). (C) Male participant’s view, seated across from Barack Obama, with Einstein avatar representing artificial intelligence present, second VR experience. (D) Virtual mirror view showing male avatar, Barack Obama avatar, and Einstein avatar.

Both findings are at the base of the VR self-conversation paradigm, first introduced by Osimo et al [6] (Figure 1). The virtual environment comprises a consultation room and 2 avatars. One avatar represents the participants as themselves, and the other represents the participants as the counselor. The participant is able to swap between the 2 avatars, thus enabling a unique experience of talking to a representation of oneself. The VR self-talk experience is based on the ability of participants to switch in and out of virtual bodies in VR. Multisensory integration between the virtual body and real body yields a strong illusion of ownership of the virtual body [7]. The virtual body moves with the participant’s movements, and the participants can see their virtual body from the first-person perspective, and in a virtual mirror. The participants (as well as their avatars) are seated, and the illusion is based on the upper body—hand and head tracking (head is visible only in the mirror; Figure 2, top).

The initial study compared 2 counselor avatars; 1 group interacted with an avatar resembling Sigmund Freud, while the other engaged with an additional avatar resembling themselves. The results indicated that using the self-talk paradigm proved beneficial in finding more satisfying solutions and improved mood, with the Freud group exhibiting better outcomes. Slater et al [7] showed that body swapping led to superior results compared to a prescripted and animated Freud virtual body and that body ownership of the Freud body was an important contributor.

Since then, these results have been replicated in multiple domains. VR self-talk has been shown to improve motivation for weight loss in individuals with obesity when they conversed with a future self who had lost and maintained weight over a hypothetical 5 years [11]. Convicted offenders spoke to their future selves, reducing self-defeating behaviors, such as alcohol consumption and violent behavior toward others [12]. An additional study showed that using VR self-talk when the “counselor” is an avatar of a leading athlete, such as Serena Williams or LeBron James, could improve adherence to an exercise regime (D Levy, unpublished data, June 2021). All these examples imply that using VR self-talk enhances cognitive flexibility, subsequently facilitating behavioral changes that previously appeared unattainable. Importantly, the design of such paradigms requires care and attention; a study using stereoscopic video revealed that besides the potential benefits of self-talk in VR, under some circumstances, the experience can also “backfire” for some specific populations [13].

Despite the success of VR self-talk, Doron Friedman, Prof Dr, and Mel Slater, Prof Dr, report (email, March 20, 2019) that people occasionally tend “to get stuck” in the conversation and cannot provide a suitable solution or advice. Sometimes, participants want to end the session or do not know how to continue after a certain point. Often, people run out of advice from the counselor’s point of view. This is where AI can play an important role. Using LLMs, we can provide individuals with the external help and ideas they need to continue making progress in their self-talk sessions. This use of AI can also be considered an intermediate step toward automated therapists.

#### Automated Dialogue

With advancements in machine learning, natural language processing, and deep neural networks, AI has become more capable of performing complex tasks and understanding human language [14]. One of the most notable developments is the emergence of conversational agents, such as ChatGPT, based on LLMs [15]. LLMs have seen tremendous advances in recent years, thanks to increased computational power and the availability of vast datasets for training. Models today, with many billions of parameters, can generate remarkably humanlike text and engage in dialogue while demonstrating some reasoning capabilities [16]. However, significant challenges remain in making these models more aligned with human values, interpreting instructions correctly, and generating factually accurate statements [17]. Current models may generate plausible but incorrect or nonsensical text. Thus, while LLMs today are impressive and valid for specific applications, they require close monitoring and oversight before being deployed into sensitive real-world settings, such as the clinical psychology domain [18].

Embodied AI applications have increasing relevance in mental health applications ranging from social robots to artificially intelligent virtual agents [19]. These applications aim to improve the quality of care and control expenditures [20]. In addition, they are also an important pathway through which the availability of therapeutic treatments may expand [21]. While AI mental health technologies continue to advance rapidly, a significant challenge remains in their successful implementation within clinical settings, where both practitioners and patients face barriers to adoption [22].

The psychological implications of the representation of the virtual therapist have been studied for decades [23], showing mixed results; sometimes, a human appearance enhanced the effectiveness of an application, while at other times, it did not. A meta-analysis by Weber et al [24] demonstrated that adding a face (as opposed to just voice or text) was more significant than the effect of realism; that is, there was greater gain in impact from having a face than from making that face more photographically or behaviorally realistic. Demeure et al [25] evaluated the impact of an agent’s representation (in a nonimmersive environment) and perceived emotion over the perceived social believability in the agent. They found that appropriate emotions conveyed through the agent’s body, mainly related to the sense of competence and warmth, could lead to higher believability.

LLMs can be the missing piece to the puzzle of VR in therapeutic mental health treatment. In this study, we examined whether LLMs can support participants in their self-talk when they run out of advice and understand how we can use an AI character to help people feel safe and not judged. We describe lessons learned during the development of this unique protocol, which involves a range of advanced techniques such as body ownership illusions [6], self-talk in VR, and LLM-based virtual humans [26]. Finally, we evaluate the final version of the system with a qualitative study.

### Iterative Design and Pilot Studies: Lessons Learned

Integrating LLM into VR self-talk requires text-to-speech and speech-to-text functionalities. To address this, we used MILO [27]. The system transcribes participant voice responses from within the VR session to text, which then serves as input for the LLM. Once the LLM generates a response, the text is converted to speech and played through the Unity application.

Initially, the AI was represented as a “help” button with voice functionality (Figure 1). Participants were instructed that a virtual counselor was listening to their conversation, and they could receive its input if they felt stuck and did not know how to continue the self-conversation. We have evaluated this as part of a study comparing VR self-talk with a physical-world equivalent setup, the empty chair technique from Gestalt therapy [28]. However, of the 11 participants in this pilot study, only 1 (9%) engaged with AI. It turned out that a short psycho-education tutorial regarding basic emotion regulation skills followed by VR self-talk resulted in an overwhelming experience; all participants were highly engaged in the session and did not remember they could ask AI for “help.” The minimal AI representation within the virtual environment, as only a button, can explain why it was ignored. In additional pilot evaluations, the combination of less-than-perfect speech recognition with less-than-perfect dialogue capabilities (refer to the information presented subsequently) often resulted in low-quality generated responses from the agent.

To make the AI agent more salient in the experience, we replaced the clickable button with a virtual human (Figure 2, bottom). This required addressing the repetition of texts; a by-product of the VR self-talk paradigm is that each text is repeated twice. First, it is spoken by the participant live and recorded, and next, it is played back by the corresponding avatar to be experienced by the participant after the body switch. When there was a third character in the scene, these repetitions became confusing. Consequently, we restricted access to the AI only from the “counselor” avatar and instructed participants that interaction with the AI avatar was recommended only between speech turns.

Furthermore, significant progress was made in LLMs over the 2 years of development. Initially, we used a specific model developed in the laboratory. This was based on the 7B parameters version of the GPT-J model [29], fine-tuned on the 2 volumes of published counseling and psychotherapy data from Alexander Street Press. The volumes are searchable collections of transcripts containing real counseling and therapy sessions and first-person narratives illuminating the experience of mental illness and treatment. The 2 volumes contain 3500 session transcripts and >700,000 utterances between a counselor and a patient. We fine-tuned the model with an 80% to 20% train-test split. While this model was state of the art at the time of the early sessions, rapid developments in LLMs rendered it obsolete; therefore, we replaced it with a pretrained model by OpenAI (described in Materials and Equipment in Methods section).

Our LLM interface provides an interface for a human operator, which can be used by the experimenters (refer to the study by Shoa et al [27] for full details). The ongoing automatically transcribed conversation appears in a text window. The operator can select parts of the text or even modify the text and send specific parts to the voice playback in the application. We have also explored the possibility of allowing the experimenter to decide when to intervene in the conversation.

To allow for a realistic experience, we designed and implemented the AI avatar to be gaze activated; that is, the virtual human representing the AI played an idle and silent animation loop and only spoke after the participant, while in the body of the counselor, stared at it for a duration of 1 second. Such gaze activation requires careful tuning—if the gaze duration is too short or the gaze area too broad, false positives might occur, whereas otherwise, the activation becomes unnatural. In our postexperimental interviews, none of the participants complained about this type of activation.

Another challenge was response latency. The delay includes the following: (1) waiting for the participant to finish talking (indicated by clicking a controller button), (2) gaze activation of the AI avatar, (3) speech recognition in the cloud, (4) latency of one LLM predication, mostly depending on server configuration and context length, and (5) cloud-based text-to-speech conversion. To mitigate latency, the system can be used in a continuous mode; that is, the system is prompted to keep generating responses after each counselor utterance based on the ongoing conversation, regardless of whether the AI agent was activated or not.

Therefore, the consequence is that latency mostly depends on LLM prediction call latency. When using laboratory-based LLMs running from our own server, we could control the latency. The overall round trip would take several seconds, and this was acceptable in the context of a counseling experience. Using OpenAI resulted in depending on OpenAIs’ response time. In our study, there were no major delays, and none of the participants in the postexperiment interview (as described subsequently) complained about latency.

Finally, we note that due to limitations in AI tools in many languages, we had to carry out the sessions in English rather than the local native language. Hence, the participants were selected to participate in the study based on their level of English speaking, ranging between conversational level English and native. Language support limitations include all parts of the pipeline: recognition, dialogue, and generation quality.

## Methods

### Overview

The system was iteratively refined and tested as described before until pilot studies indicated that it operated smoothly. Consequently, we carried out a qualitative explorative study.

Because the overall experience was very rich and overwhelming (as described subsequently), we opted for an exploratory study, carrying out careful sessions, including the presence of a trained clinical psychologist. Although it was made clear to the participants that the system was experimental and this was not a real counseling session, it was clear from the responses that participants behaved almost like in a “real” counseling session, and most participants went through a psychologically meaningful session, as evident from in-depth postexperiment interviews (refer to the Results section).

### Participants

The study population comprised 14 anonymous university students (female participants: n=5, 36%) aged 19 to 26 years (mean 23.43, SD 2.21 years). Participants who self-declared as English speakers, had no history of epilepsy, and were not pregnant at the time of the study were invited to participate in the study as part of their credit requirements for their BA psychology degree. In total, 3 (21%) of the participants were not included in the final sample due to miscellaneous technical problems that came up during their session. Given the limited sample size, data saturation was not reached.

### Materials and Equipment

#### Overview

The VR environment featured a consultation room. The VR simulation was developed with the Unity game engine (version 2020.3.20f; Unity Technologies). The VR headset Quest 2 (Meta Platforms, Inc), including its handheld controllers for embodiment (upper body tracking), was used. Quest 2 has a single liquid crystal display panel for each eye, with a display resolution of 1832×1920 pixels. The refresh rate of the panel is 120 Hz. Its weight is approximately 500 g, and it has a head strap that ensures comfort during prolonged use. In addition, Quest 2 delivers a comprehensive 6 dfs, providing participants with both rotational and positional tracking capabilities.

The software includes a VR self-talk experience and integration of an LLM-based agent based on MILO. In the exploratory study, we used Barack Obama and Michelle Obama, sex matched, as the counselor avatars. The AI avatar was Albert Einstein. The participants were initially embodied in a generic sex-matched avatar (Figure 2). The first part of the VR experience was intended to strengthen the virtual body ownership illusion. The participants engaged in a short “embodiment” exercise, alternately moving both hands, looking around, and seeing themselves in the mirror. Following the embodiment exercise, the participants were asked to describe a current life issue. When they finished, the participants pressed a button, transitioning them to the counselor avatar. Previous studies found that it is best to model the counselor avatar based on famous inspiring persons; we selected Barack Obama in our studies. Next, the participants pressed a button and listened to a replay of what they just said, spoken by the avatar representing themselves as patients. A prompt instructed them to respond “like a counselor,” and their responses were recorded. The participants’ voice pitches were changed in the recording to avoid having it sound exactly like the participant, as described in the study by Osimo et al [6]. Once completed, participants returned to their look-alike avatar to continue the conversation in this iterative manner.

We used 2 VR self-talk implementations; one was a commercial product called ConVRself (Kiin Tech) used during the iterative design, and the other was a research version with very similar design and functionality used during the study.

For LLM, we used GPT-3.5 using the following prompt: “You will now act as a motivational interviewer with a lot of experience in interpersonal psychotherapy. I am a counselor and will turn to you for advice while speaking to my patient. Sometimes, I will get stuck and address you, and in this case, you should try to act as an expert counselor and say something that would help me progress the session as best as possible. Please do not mention that you are an artificial intelligence in your replies. Act as a real person.”

#### Semistructured Interviews

Participants’ experiences and thoughts regarding the AI were assessed using semistructured interviews that lasted approximately 15 minutes, and the audio of the interview was recorded. Due to the exploratory nature of this complex experience, we opted for the flexibility offered by semistructured interviews. We constructed 14 questions in advance, such as, “Please describe this experience as if you were describing it to a friend who was not here,” (Multimedia Appendix 1). The questions were presented individually to the participants, and additional questions were added based on the participants’ responses. The interviews were conducted by one of the main authors (MZ), a female clinical psychologist and PhD candidate, with appropriate training. There was no relationship between the interviewer and the participants before the study commencement, and the participants were informed of her professional expertise.

The actual conversation of the participants was logged by default as part of the system, including the AI comments. However, we opted not to analyze the conversations due to the privacy of the participants, and the content was removed.

Applied thematic analysis identifies and describes implicit and explicit ideas within the data, consequently linking them into the theoretical framework of the study [30]. The analysis was thus conducted using a bottom-up approach in which the answers provided by the participants determined the main themes rather than the questions asked. In addition, at the start of the interview, participants were asked to provide a description of the experience, to elicit unconstrained participant impressions.

### Ethical Considerations

The ethical considerations regarding implementing AI in psychological settings are paramount [31]. The development process and exploratory study outlined in this paper aimed to address preliminary questions concerning effectiveness and validation. In addition, in our study, a clinical psychologist provided professional oversight, which is typically recommended for AI implementation. The study received ethics approval from the institutional review board of Reichman university (P_2022145). Participants received course credit for their participation in the study and gave written informed consent. All data protection procedures were adhered to. All participants provided informed consent, which explicitly outlined that their responses would be recorded and kept as part of the study data but would be saved anonymously and that they would interact with AI during the study, receiving advice from it regarding a personal problem.

### Procedure

Upon arrival at the laboratory, the participants were given instructions about the experiment and completed a consent form. They were told that they would have the opportunity to talk about a personal problem that caused them an average level of distress (on a scale of 1-10, a problem rated between 4 and 7). Then, they wrote a short sentence describing the problem in their words. Importantly, although it was made clear to the participants that the session was experimental and should not be considered psychological counseling, our sessions were carried out in the presence of a trained clinical psychologist in a private laboratory setting in which only the participant and the psychologist were present. Next, the participants donned the VR headset and performed the VR experience in which they conversed freely between their sex-matched avatars and matching Obama characters (Michelle for female participants and Barack for male participants). After a back-and-forth conversation between the participant and Obama avatar, the participants chose when to end the session.

After the first session, the participants answered two questions regarding their experience as follows: (1) Please rate this experience on a scale of 1 to 10 (1=not useful at all and 10=very useful) and (2) On a scale of 1 to 10, how helpful was this self-conversation for solving your problem?

Next, it was explained to them that they would perform a similar session, only this time, another third avatar, depicted as Albert Einstein, would be seated in the virtual consultation room. They were told that it would be listening to their conversation and that they should turn to him during the conversation to receive his input but only from the embodied counselor’s perspective. The gaze activation was explained, and they were instructed to turn to Einstein only from the Obama avatar. The participants proceeded to conduct the AI-enhanced iterative VR self-conversation, and when they were done, the semistructured interview was conducted.

## Results

### Overview

A total of 5 (45%) of the 11 participants chose to speak about a problem related to work or school. Another 5 (45%) participants chose to speak about relationship problems, and 1 (9%) participant chose to speak about difficulty managing stress.

The semistructured interviews were transcribed and analyzed using the thematic coding method by 2 of the coauthors. The transcripts were not reviewed by the participants. A total of 5 (45%) participants preferred the session with the AI, 4 (36%) preferred the session without it, and the remaining 2 (18%) did not address this. In total, 7 (64%) participants said that the AI’s attitude toward them was positive, 1 (9%) said it was neutral, and the remaining 3 (27%) did not address this.

The participants were asked 2 questions in the break between the first session (without AI) and the second (with AI). On average, the usefulness of the session was rated 6.9 (SD 0.54); the median value was 7 (IQR 7-7). On average, the degree to which it helped solve their problem was rated 6.1 (SD 1.58), with a median value of 6 (IQR 6-7). At the end of the interview, the participants were asked to rate the extent to which they would like to come back for another session on a scale of 1 to 10; the mean response was 8.3 (SD 1.55), and the median was 8.5 (IQR 7-9), indicating a very high level of satisfaction from the experience.

The applied thematic analysis qualitative analysis revealed 4 high-level themes, which were derived from the data. These 4 themes are outlined subsequently. The participants did not give feedback on the findings outlined below.

### Quality of Advice

From the thematic analysis of the participants’ responses, a layered or complex pattern emerged regarding the perceived quality of AI-generated advice. On the one hand, participants acknowledged the AI’s ability to provide insightful, well-structured guidance:

...Einstein brought a lot of emotional considerations to the two-way problem I didn’t think about. It was interesting...It sounds like if I would listen to him like everything would work out.

S6

For my questions it was very, very beneficial. It was. It was able to provide very thorough and clear and insightful points and information regarding specifically related to my problem related to the career path and choosing a career path. And it was really cool to see how the interaction with the computer algorithm was able to really contribute to my own self-reflection.

S8

They viewed the advice as emotional while sustaining a thorough analysis of the situation. However, sometimes this very sophistication created a feeling of uncanniness, with participants noting that it was “inhumanly smart.” Some participants pointed toward the notable tension between the logical precision of AI and the inherently “messy” nature of emotional problems, suggesting that the AI’s responses tended to be overly orderly or systematic:

I think that on the one hand it was good, and he was saying smart things, but on the other hand it was a little bit too logical and technical, and sometimes emotional problems are a little more complex.

S4

...Good as I said, but it was a little bit surface level.

S7

Like robotic smart. Like human, but inhuman smart. You know what I’m saying? Like he was too good of a psychologist. I don’t know how to say it. It felt like a nice thing to say, but feels like I don’t know how to describe it too, like, kitsch...The AI surprised me...Yeah, the AI was kind of freaking yeah. Like it felt. It felt like too good. I don’t know how even to describe it...Yeah, but also, I mean it was like in between. It was like really compassionate and understanding and like what the perfect answer from a psychologist should be. But the perfect answer is like not, not human to some degree. It also felt like he just blabbered a bunch of information, like instantly to me, like, Oh yeah, it’s OK to be compassionate and it’s OK to be. You shouldn’t be perfect all the time, like, in one sentence.

S13

There was also evidence pointing to potential overtrust in AI responses, which has been mentioned before [32]. This might suggest that careful consideration of AI authority is necessary in therapeutic settings due to increased credibility attributed to AI-reorganized versions of the participants’ own thoughts:

I think he just had a way of wording the same ideas that I had in my head, but sort of organizing them to a more sophisticated or organized manner which made them more believable or reliable, seem more reliable.

S11

In addition, some of the “complaints” could also be a reasonable approach to a human counselor:

S6: ...that it was not really practical, but it was like another part that you need to think about.

Question: What wasn’t practical about it?

S6: Like for my problem. I need an answer, it’s like a yes or no question and he really talked about like what it made you feel and maybe rephrase the problem, and there’s no way to really rephrase it.

### Self-AI Collaboration

In some cases, participants highlighted the complementary nature of the combination between the AI advice and their own input, suggesting a unique form of alliance where AI and human intelligence united and enhanced each other’s strengths. The AI’s tendency to a more structured approach was balanced effectively with the more emotionally attuned self-counsel, creating what one participant even termed as a “perfect” mixture. This combination led to a more comprehensive experience. As mentioned, rather than viewing AI as a replacement for traditional therapy, its optimal role, as the participants experienced, was as a tool that enhanced their self-reflection and personal insight:

...I liked the mix of the AI and myself as the counselor...I think the advice I gave myself was taking into account more like an emotional feeling and maybe even a little bit too much. And he was a little too technical. So maybe a mixture of them? Yeah, a mixture of them would be perfect.

S4

I think in terms of advice, it was similar to what I said, although the AI was able to provide a more detailed and well-structured answer. The AI’s examples and ideas were a bit more beneficial.

S8

And I liked the mix of him and Michelle together, that I, as Michelle gave a bit of a softer input and he was like, do this and this and this and this and that together was like, they filled each other...No, I think they were really complementary to each other. I benefited very much from both of them. I’m really grateful. I got to talk to myself...OK, more minds better.

S9

### Believability and Attitude Toward AI

The analysis suggested a notable dichotomy between the cognitive aspect and the perceptual issues of the experience. While the content and reasoning capabilities of the AI were valuable and convincingly humanlike, the nonverbal and visual elements created “breaks in presence” [33]. These elements were mainly manifested in voice quality, robotic movements, and lack of natural pauses in speech turns:

...yeah, the sound and obviously the, the graphics obviously. But in terms of the syntax and the structure of how the answers were provided and delivered then it was fairly good. Fairly satisfying.

S8

I feel like a mixture, like the words that he was saying felt like they made sense, they were human-like, but the way that he was telling them and like it was very technical...

S4

The conversation felt like very human and Einstein Avatar was a little bit robotic and felt like scripted and just a little bit like not related...No, the look was OK.

S5

I’m asking a question and then we’ll just speak like we talk and talk and talk and talk...Humans usually wait a little bit.

S7

Technical stuff, which is like the character will move a little bit differently than human, and that’s pretty much.

S7

However, a phenomenon of gradual acceptance regarding the participants’ ability to adapt to the limitations of technology aligned with established research on place illusion in immersive VR environments [34]. In other words, the participants wanted to believe what they were experiencing in such a way that they were willing to ignore the technical imperfections that created disbelief. This process of adaptation was evident in the participants’ ability to shift their focus from the robotic characteristics to the contextual value of the dialogue. In this way, the content served as a compensatory factor, allowing participants to overcome initial reservations regarding the artificial nature of the interaction. While technical refinements and improvements in nonverbal behavior are called for, the therapeutic value of the AI-advice might be more dependent on the quality of the dialogue rather than the behavioral human mimicry:

I think it’s still not 100% human like but it did feel like I was talking to a very insightful person because at the end of the day I was I was focusing more on the information that was given to me by the algorithm rather than the actual feeling and and the the sound of the the interaction itself.

S8

...at once I got over the metallic voice, yeah, it felt a bit more natural...It’s like a fake character. It’s an avatar, but I think when you get in, in into it, it’s become less and less weird. Less and less, Yeah.

S9

### The Use of Celebrity Avatars

The data suggest that the selection of well-known figures as therapeutic avatars creates complex psychological dynamics that can either enhance or impede the experience. Participants’ reactions demonstrated that the personal associations with these celebrity avatars substantially influenced their engagement with the AI and with their own counselor persona. For instance, responses to the Obama avatar highlighted the importance of perceived authority and strength, while reactions to Einstein often centered on his scientific reputation and intellectual credibility. However, these preexisting associations could also act as barriers, with some participants expressing a preference for avatars they could “relate to and love more.” This finding suggests that the effectiveness of celebrity avatars in therapeutic settings may be highly individualized, with the same figure potentially eliciting different responses based on personal preconceptions. The data particularly emphasized how the avatar’s identity can affect the reception of therapeutic advice, with some participants explicitly attributing the perceived value of the guidance to the celebrity’s reputation rather than the content itself:

And I think Obama is quite a good character to use, actually. I feel that she’s confident. She’s a woman, I guess like a strong woman. She’s very powerful. It seems like it’s very fun to sit around her and like look at her and talk to her.

S5

That’s the point, Talking to Obama, even if, like, I don’t agree with him that much. But like, yeah, it’s a cool person to talk to.

S7

I think I would change Barack Obama’s character to someone that I love. As I said, one of the reasons I felt more comfortable with Einstein is because that’s a character I can relate to and love more.

S10

Again, maybe it’s just because it’s Einstein. I mean if like a human being would say something like that to me but would phrase it differently and, you know, have different facial expressions like not like the computer Einstein, then I would feel differently about the answer.

S13

...Maybe because it was Einstein. Like maybe if it was someone else maybe I wouldn’t think about like it was but just because it was him and I know that he was like really big scientist so he might have like great things to say.

S14

## Discussion

### Principal Findings

Mental health and well-being are major challenges worldwide, and the demand for counseling is much greater than the supply. Mental health systems worldwide are under immense strain, and in the face of growing need, many underqualified or pseudoprofessional services have appeared. As a result, an increasing number of stakeholders are turning to AI-based solutions, including LLM-driven tools, as a potential quick fix (this includes Israel’s minister of science [January 3, 2025, personal communication]). However, we advocate a more measured approach, emphasizing rigorous evaluation and clear delineation of clinical versus nonclinical use. Our intervention explicitly targets nonclinical populations (ie, focusing on mental health improvement rather than formal therapy) and relies on AI only as an adjunct to self-talk rather than a stand-alone therapeutic agent. Such caution is vital given the nascent state of these technologies and the ethical implications tied to safety, efficacy, and user well-being. Accordingly, we strongly support further research and stepwise validation, coupled with the ongoing involvement of trained professionals before any large-scale implementation.

Technologies, such as VR and AI, may be a part of the solution. VR self-talk is unique in suggesting counseling without the need of either a human in the loop or AI and has been shown in the past to be beneficial and effective. Adding an LLM assistant is a natural next step; nevertheless, introducing an LLM-based AI counselor assistant into VR is not trivial, and our iterative design process has revealed several important lessons.

Integrating multiple challenging technologies, such as VR, body tracking, dialogue, voice recognition, and speech generation into a meaningful psychological experience proved arduous. While each of these technologies separately has made impressive progress recently, accumulated problems or errors easily resulted in an unusable experience. In the case described here, it seems that during 2023, the underlying AI technologies, specifically speech recognition and LLM-based dialogue, passed an essential threshold, increasing the probability of a successful and meaningful experience regardless of the complex nature of the technological design.

While both VR and AI, individually and in combination, present significant opportunities for counseling, their widespread deployment in real-world settings remains challenging [35]. The contribution of each of these components as well as their combined contribution needs to be carefully evaluated [36]. Industry trends indicate that integrating AI models into a wide range of applications is becoming viable, for example, applications such as Tess and Woebot [37]. VR is gradually becoming more accessible, though its availability remains somewhat limited. We propose that the experience of “interacting with yourself in the third person,” facilitated by VR and virtual body–ownership illusions, offers unique benefits that are unlikely to be replicated on 2D screens. In addition, the integration of LLMs into text and voice-based interactions is expected to become more widely accessible. Therefore, we hope that this study, despite its challenges and complexities, can serve as a foundation for further advancements in this field.

On the basis of the themes that emerged from the semistructured interviews, we can conclude that participants generally responded positively to the AI advice, with reactions ranging from mixed to favorable. None of the participants evaluated it as poor or as obstructive to the self-talk process. Considering the complexity of the experience, this result is very encouraging. VR self-talk, when performed correctly, is a very powerful experience, though it can be confusing even without the addition of AI. To address this, we implemented a gradual protocol, allowing the participants to accustom themselves to the VR self-talk before introducing the AI agent in a subsequent session. This protocol was necessary for our preliminary research. However, future studies could benefit from a research design such as randomized controlled trials that could compare self-talk with and without AI, providing a deeper understanding of the intricate nature of this experience and the added value of AI to it.

The overarching goal of this endeavor was to design and implement an AI agent within a self-talk experience. We envisioned the AI advice as complementing the participants’ own self-guidance rather than replacing their need to think, reflect, and reason with themselves regarding the issue at hand. This vision was in alignment with the participants’ responses. They found it straightforward to implement the AI advice into their self-dialogue and quickly adapted to the flow of the conversation. Our results further support the perspective that the integration of technology into counseling has the potential to enhance skills and abilities that humans already possess [16].

In our case, an “AI” button was not enough, and we opted to integrate a gaze-activated avatar to embody the AI. While participants commented that the voice and animation of the avatar were not completely realistic, several of the participants indicated that they were able to overcome such limitations and focus on the social interaction as well as the content of the conversation.

Finally, most of the participants pointed to the need to improve design elements, such as voice, body language, avatar appearance, and character selection. There was a relative consensus among participants regarding the need for improvements in this area to enhance the overall experience. This is not surprising as the level of realism has been shown to have a strong impact on affective responses of participants [26,27]. Now that we have successfully integrated multiple technologies and created a seamless psychological experience, future development and testing should focus on refining and improving these design aspects.

### Limitations

This study has several limitations that should be addressed. Although multiple pilot studies were conducted throughout the iterative design process, including approximately 30 additional participants, the final sample consisted of only 11 participants. The relatively small sample size underscores the necessity for further research with larger and more diverse populations that could confirm these initial results. However, as emphasized throughout this paper, this is a novel and promising application of technology, and every exploration must begin somewhere. Due to the psychological focus of this study, further research should explore specific participant characteristics that could influence the effectiveness of self-talk in VR. Furthermore, research could target particular symptoms of psychopathology or specific diagnoses to improve the understanding of potential clinical applications.

Another limitation is the bias of novelty. The use of VR and AI in a psychological setting could have potentially fascinated the participants, leading to an inflated sense of efficacy and distorting the true impact of the experience. In addition, semistructured interviews could have contributed to social desirability and acquiescence bias, as participants might have felt inclined to provide responses they expected would be favorable to the researcher. Further research could explore ways to mitigate these identified biases.

Technological limitations are the lack of systematic analysis of the model’s responses and their adherence to the therapeutic principles outlined in the prompt. The quality of the model’s response is influenced by both the prompt and the internal architectures of the LLM itself, in our case GPT-3.5, which has proven highly capable but is proprietary and continuously evolving. The initial study used a relatively simple prompt, instructing the AI to adopt the role of an experienced motivational or interpersonal psychotherapist. While this approach was based on established research [38], it did not undergo formal pilot testing specifically targeting the fidelity of the model’s alignment with the therapeutic technique. This decision was made, in part, due to the exploratory nature of this study, which aimed to assess the potential use of LLMs in VR-based self-counseling rather than conduct an in-depth evaluation of their adherence to specific therapeutic techniques. Nonetheless, we acknowledge that future work would benefit from piloting more refined prompts, including detailed instructions for eliciting key therapeutic elements.

The field faces significant challenges in ensuring replicability and consistency, particularly when relying on such models. As a result, the research community must consider how to establish more robust methods for quality control and model evaluation to ensure the ongoing utility and ethical deployment of LLMs in therapeutic settings. Recommendations include clinical evaluation of the LLMs developed and their output; interdisciplinary collaboration; and attending to risk assessment, transparency, and bias [36]. We were able to implement some of the recommendations in our initial study, such as clinical evaluation of output and interdisciplinary collaboration, and further studies could improve on others.

### Conclusions

The introduction of new technologies could potentially transform psychotherapy, giving rise to numerous potential challenges, limitations, and ethical considerations that should be addressed. Currently, clinical expertise is necessary for best practice; however, that may evolve in the future, and even now, monitoring and validating can be more cost-effective for certain patients compared to traditional psychotherapy. Given our encouraging results, we suggest that the paradigm of AI-enhanced VR self-talk may be ready for further research on a larger sample from the general population and studies with clinical populations. In addition, our work suggests there are numerous potential opportunities for integrating AI into VR wellness, extending beyond just “automated therapist agents.”

## Appendix

Multimedia Appendix 1 Complete interview protocol used in this study.

## Abbreviations

AI: artificial intelligence
LLM: large language model
VR: virtual reality

## Data Availability

The datasets generated or analyzed during this study are not publicly available due to the confidential nature of the content, but numerical results will be made available from the corresponding author on reasonable request.
