# Supplementary material for: AI-Enhanced Virtual Reality Self-Talk for Psychological Counseling: Formative Qualitative Study
Source: JMIR Form Res. 2025 Apr 2;9:e67782. doi: 10.2196/67782 (PMC12004015; doi:10.2196/67782)
Supplement: Multimedia Appendix 1 [file formative_v9i1e67782_app1.docx]

Hi, and thank you for participating in our study on the effects of self-conversation on problem-solving. You will experience two virtual reality (VR) sessions. Soon I will help fit the headset, and you will see an avatar of a man in front of you. Please use the arrows on the top of the screen to choose between the male or female avatar. You will be seated across from an Obama avatar.

When you press the right trigger, you can explain your problem. After finishing, press the right trigger again to swap into Obama's body. You’ll hear your explanation, and then respond to yourself on how to address the issue. You will have a self-conversation, swapping back and forth between both avatars, please feel free to talk as much as you want. Please select a current personal problem that causes moderate distress, rated between 4 and 7 on a scale of 1 to 10.

Please sign the consent form and answer the gender and age questions. Additionally, write down the problem you plan to discuss.

Run VR app Obama1.

Questions for between the first session to the second:

1. Please rate this experience on a scale of 1 to 10, 1=not useful at all, 10=very useful
2. On a scale of 1 to 10, how helpful was this self-conversation for solving your problem?

Turn on Python (view video). Write the participant id.

Run VR app Obama2.

The second VR experience is the same but includes a facilitator. You can look to the facilitator for input regarding your problem while embodied as Obama. When you look towards the facilitator, depicted as Albert Einstein he will start to talk and give you advice regarding the problem at hand.

Fit the headset for the participant again.

General opening questions-

1. Please describe this experience as if you were describing it to a friend who wasn’t here.
2. What were your expectations?
3. Was there anything about the experience that surprised you/exceeded your expectations? (about the self-conversation/AI)

About the AI

1. Can you tell me briefly about your thoughts regarding the facilitator?
2. How easy/difficult was it to engage with the facilitator? (Did it feel natural/humanlike or not?)
3. What was the facilitator's general attitude towards you? (neutral/negative/positive)?
4. What did you think of the facilitator's advice?
5. How would you compare it to the advice you gave yourself?
6. Did you prefer the session with or without the facilitator? Did one session feel more helpful than the other?
7. What part of the experience was beneficial/had an impact?
8. How did it make you feel?

Additional impression-

1. If you could change one thing about the experience, what would it be?
2. Is there anything else you would like to add?
3. On a scale of 1 to 10, can you rate if you would like to come back for another session (1 signifies not at all, 10 signifies very much).
